# A Systems Thinking Approach to Understanding and Demonstrating the Role of Peer-Led Programs and Leadership in the Response to HIV and Hepatitis C: Findings From the W3 Project

**DOI:** 10.3389/fpubh.2018.00231

**Published:** 2018-08-31

**Authors:** Graham Brown, Daniel Reeders, Aaron Cogle, Annie Madden, Jules Kim, Darryl O'Donnell

**Affiliations:** ^1^Australian Research Centre in Sex, Health and Society, La Trobe University, Melbourne, VIC, Australia; ^2^School of Regulation and Global Governance, Australian National University, Canberra, ACT, Australia; ^3^National Association of People Living With HIV Australia, Sydney, NSW, Australia; ^4^The Australian Injecting and Illicit Drug Users League, Canberra, ACT, Australia; ^5^Scarlet Alliance, Australian Sex Worker Association, Sydney, NSW, Australia; ^6^Australian Federation of AIDS Organisations, Sydney, NSW, Australia

**Keywords:** systems thinking, peer education, peer leaders, community, HIV, Hepatitis C, leadership, evaluation

## Abstract

The central role of community and peer-led programs has been a key characteristic of the Australian partnership response to HIV and hepatitis C since the beginning of the epidemics. Despite this, peer-led programs continue to have limited capacity to demonstrate their role and value as part of a multi-sectoral response. What makes one peer-led program a better investment than another? What role does the rest of the sector have in ensuring we gain the most value from these investments? To investigate this, we facilitated interactive systems thinking methods with 10 programs working within communities of people who inject drugs, gay men, sex workers and people living with HIV across Australia. This included articulating program theories in diagram and textual form to help us understand the role of peer-based programs promoting peer leadership within the Australian HIV and hepatitis C responses. Our aim was to develop a framework for monitoring and evaluation that could be applied to peer led programs at different levels and in different contexts. We found that for peer-led programs to fulfill their role, and to navigate the rapid changes occurring in the both epidemics, they need to: demonstrate the credibility of their peer and community insights; continually adapt to changing contexts and policy priorities in tandem with their communities; and maintain influence in both community and policy systems. We developed a framework of four key functions (Engagement, Alignment, Adaptation, and Influence) which peer-based programs need to demonstrate, which form the basis for identifying quality indicators. This article presents a new way of framing and monitoring investments in peer-led programs and peer eadership actions by these programs. If health policy is committed to strengthening the leadership shown by affected communities, then we need to understand, enhance, monitor and value the role of peer-led programs and peer leadership within the overall prevention system. We believe the W3 framework, drawing on systems thinking and modeling, can support funders, policy-makers and programs to achieve this.

## Introduction

Australia has a long history of a partnership response to HIV and hepatitis C, comprising a collaborative network of policy jurisdictions, health services, community organizations, and researchers. However, these networks are now havin to adapt to the largest and most rapid changes in the nature of these epidemics for decades. These changes include unprecedented developments in prevention and treatment technologies, including major policy and practice shifts to prioritize the use of treatment as prevention in HIV and HCV (1–3). This is where people infected with the virus are encouraged to seek treatment for both their personal benefit and for the broader public health benefit of reducing viral transmission (4, 5). This growing prominence of clinical interventions and services for prevention is accentuated further in HIV, where drugs used to treat people living with HIV are now being used by people without HIV to stop them acquiring HIV—known as pre-exposure prophylaxis (PrEP) (6).

The social, sexual and injecting norms and practices of communities of gay men and people who inject use drugs are already adapting to these changes—well in advance of policy, health systems, and public health responses (7–9). For example, in many countries PrEP (10, 11) and treatment as prevention (TasP) have profoundly disrupted meanings of safe sex and the way HIV stigma is resisted or reinforced. It has seen the emergence of community-initiated access schemes based on private importation and advocacy for publicly-funded access to medications (12). Uptake of the new technologies has disrupted the delineation between community services and health services and community workers and clinicians (13, 14). The community, the peer-led programs and the health and policy system are needing to adapt and change in tandem with each other in an environment that is constantly in flux.

Peer-led programs are led and conducted by people from the communities most affected by HIV and hepatitis C, and operate through organizations established and governed by these communities. These programs may include peer activities ranging from peer service delivery (such as peer-led rapid HIV testing or peer-led needle and syringe program), to peer health promotion (such as peer developed and implemented campaigns or community development), to peer leadership (such as peers taking leadership roles in their community, their sector, or participating in policy and law reform). Peer leadership is not confined to the management positions of peer organizations, but refers to the contribution these programs, through a range of activities, make to policy-making and strategy development, either through. This can include, community empowerment and mobilization, direct advocacy or by training and supporting individual advocates, participation in decision-making, monitoring of responses, and participation in community-based research (15, 16). One of the factors which hinders peer led programs from maximizing their potential role is their limited capacity to monitor and demonstrate their quality and effectiveness. The effectiveness of peer-led programs depends on their ability to adapt quickly within a complex and continually changing environment.

Peer led programs are expected to advocate for the needs and experiences of an increasingly diverse and dynamic group of people within a policy and political climate that is constantly changing and contested. In this complex circumstance, on what basis can we assess the quality and effectiveness of peer leadership activities of a program–and what role do other policy actors play in gaining the full potential value from the investment in it?

Monitoring the quality and impact of specific peer-led programs and their leadership role is challenging (17–21). Most evaluations look at the quality and impact of programs in isolation from their context and measure fidelity to a pre-defined protocol, and therefore struggle to capture the complexity of their interaction with and adaptation to the rapidly changing community and socio-political contexts in which they are implemented (22). However, community and peer-led health promotion is *all about* interactions between the program, the communities they work with, and the policy environment within which they operate and collaborate. What this often leads to is a policy rhetoric about community mobilization and peer leadership and the importance of combining behavioral, biomedical, and structural responses, but community and peer-led programs being funded, monitored and managed as standalone instances of generic interventions. There is conflicting evidence as to the cost effectiveness of peer led health promotion (23–25), however often omitted from the analysis is the potential community and policy system roles of peer-led programs. Moreover, cost-effectiveness studies tend to focus on direct effects rather than synergistic effects registered in other parts of the prevention system as a result of the contribution made by peer leadership activities and programs.

The What Works and Why (W3) Project took a bold new approach to develop a practice based theory and evaluation framework for peer-led organizations, including peer leadership, to monitor and demonstrate their role and influence. Our aim was to develop a framework for monitoring and evaluation that could be applied to peer led programs at different levels and in different contexts. We facilitated complex systems thinking methods workshops (26, 27) with 10 programs working within communities of people who use drugs, gay men, sex workers, and people living with HIV across Australia to understand the role of peer-led programs within the Australian HIV and HCV response. This article will draw on the findings of the W3 Project to present what we believe is a more relevant way of framing and monitoring our investments in community mobilization and peer leadership, and discuss the implications for peer-led organizations and the broader HIV response.

## Methods

To develop a planning, monitoring and evaluation framework based on insights from peer-based practice, we applied a systems thinking approach that conceptualizes peer-led programs, and the communities and policy environments they engage with, as complex systems. Systems thinking helps us to identify and understand the complex relationships between all the moving parts of a community and policy system, while complexity science shows how interactions among actors can generate emergent structures (such as networks, cultures and communities) and effects (including overall prevention efficacy). The approach aims to simultaneously consider the big picture, the individual pieces that make up the picture, and the complexity of non-linear relationships and emergent effects (28–30). What systems thinking brought to the research was an understanding that for peer-led programs and their leadership–the ways in which communities engage with, enhance, adapt, resist or disrupt interventions are part of the program and something to be leveraged, not resisted or treated as a confounder (22). We felt that the systems approach could bring our understanding of peer-led programs and their role in peer leadership closer to the reality of these programs on the ground.

A system thinking approach recognizes that peer-led programs, community action, policies, laws, culture, health systems and new technologies will interact and influence each other, whether this is planned or not, and these interactions can produce important but unpredictable effects that can help or hinder prevention. Thinking about prevention as the outcome of complex system directs our emphasis toward identifying leverage points and synergies within the workings of that system (31). Systems thinking is an emerging field in public health and has seen uptake in other complex health and social challenges such as obesity (32–36), tobacco (37), as well as other areas (38–40). While the approach has been advocated by WHO (41), it has had limited application to practical use within community based HIV responses (42–44).

The study was conducted over four phases:

The first phase conducted a series of highly participatory workshops with the partner organizations to develop system maps. We then analyzed the system maps (Phase 2) to identify common themes and functions, and develop a draft framework. We then subjected this draft framework to review from non-partner organizations and stakeholders from across Australia (Phase 3). We then worked with the partner organizations to apply the W3 Framework to identify quality and impact indicators (phase 4). These four stages are described in detail below.

### Phase 1—participatory workshops to develop system maps

Over a 2-year period, the W3 Project conducted a series of 18 workshops, ranging from 1 to 2 days each, with peer-led organizations working with gay men, people who use drugs, sex workers, and people living with HIV in Australia (see Table 1). Some workshops were with single organizations and some with up to four organizations. All the participating organizations conducted peer-led programs with a mix of paid and volunteer staff. The size and capacity of the organizations varied significantly (ranging from four paid staff to over 40). All were engaged in peer service delivery, peer health promotion and peer leadership activities to varying degrees. Organizations participated in the project on the basis that staff participation in workshops was voluntary. Each participant in the workshops was provided a plain language information pack about the project and provided signed voluntary informed consent to participate in each aspect of the research data collection. The project was provided ethics approval by the La Trobe University Human Research Ethics Committee (Approval No: FHEC14/155). More than 90 staff and volunteers were involved across the workshops (45).

**Table 1:** The W3 collaboration.

Australian Federation of AIDS Organisations (the national body for the community based response to HIV), www.afao.org.au Australian Injecting and Illicit Drug Users League (the national body for peer-led based drug user organizations), www.aivl.org.au Harm Reduction Victoria (peer-led drug user organization), hrvic.org.au Living Positive Victoria (PLHIV peer-led organization), livingpositivevictoria.org.au National Association of People Living with HIV/AIDS (national peer PLHIV organization), napwha.org.au Positive Life New South Wales (PLHIV peer-led organization), www.positivelife.org.au Queensland Positive People (PLHIV peer-led organization), www.qpp.org.au Scarlet Alliance – Australian Sex Workers Association (national peer-led sex worker organization), www.scarletalliance.org.au Victorian AIDS Council (community and peer-led organization), vac.org.au Western Australian Substance Users Association (peer-led drug user organization), www.wasua.com.au

*In Australia “community based” and “peer-led” and are the dominant organizational descriptors. These organizations were established by the communities most affected by HIV from the mid-1980s, and their governance is based within their communities. The majority of their limited funding comes from national and state governments with varying contracting conditions and caveats. Community based organizations at a policy level are considered part of the “HIV partnership” alongside clinical services, research, and government. As with most countries, these relationships have waxed and waned over the past 3 decades. For a summary of the history of the community response in Australia see (55)*.

We used systems thinking approaches to elicit and diagram mental models of how peer-led programs operate from the experience and perspective of peer practitioners working in outreach, community development, workshop facilitation, policy reform and leadership, management and governance–recognizing that everyone has a part of the picture but the pieces are rarely brought together. In other words, we drew on implicit theories embedded in practice (46) as primary data to construct explicit program theories (47) of how peer based programs work.

The methods for the workshops were as follows. At each 2-day workshop, we shared a hypothetical narrative of an event that had been drafted in consultation with liaison staff at the partner program to highlight differences between a peer and non-peer model of service. The narratives were discussed in small groups. A modified version of Maani and Cavana's “iceberg model” (48) was used to frame questions for the small group discussions, and researchers and participants scribed brief insights from this discussion onto Post-It notes.

In its original format Maani and Cavana's (48) “iceberg” model invites reflection on events, patterns, structures, with mental models at the base. In our version of the model we asked about events, patterns, structures and *cultures*—since cultures are how mental models are transmitted and inculcated in a peer networks or communities. Facilitating the discussion using the iceberg model made it possible to reconstruct, from the mental models that different participants described to understand and explain the hypothetical event depicted in the narrative, how the program learns about the social systems it engages with. Following the small group discussions to generate insights, affinity methods were used to discuss different ways of grouping the insights to identify higher-level themes.

This provided a structure for facilitated small group discussions, and insights from these discussions were recorded onto Post-It notes. Whole group discussions were then used to discuss different ways of grouping the insights into higher-level themes.

Drawing on complex systems theory and methods (26, 27, 49, 50) we developed causal loop diagrams (CLD) (26, 27), a tool used in systems thinking to identify the regular (i.e., cyclical) relationships or “loops” among the higher-level themes. They provided a visual language for specifying relationships and flows of influence, thus helping us theorize what social processes and causal mechanisms might underlie the repeated emergence of these themes in program and sector-level discussions. For example, a commonality among the diagrams was that flows of *knowledge* were essential for the purposes of guiding adaptation, both within the program and the sector, to maintain sustained influence within both the target communities and the policy context.

On the second day of the initial workshop the draft CLD was demonstrated and tested by picking an issue of concern for the peer program, and after selecting a starting place on the map, followed along the pathways to invite discussion of how the issue might “play out” on the system depicted. We asked participants to tell us what variables and relationships were missing and what needed to be changed. We simulated the map in discussion by asking “what would happen if x (item) changed drastically?” These discussions repeatedly generated identification of items that considered important in practice but missing on the map, or that were conceptually necessary within the emerging logic of the map. Subsequently, the research team in consultation with partner liaisons identified causal loops and longer multi-loop “pathways” among the items on the maps. We articulated the strategic implications of these structural features in text, and presented them to participants and stakeholders for reflection and discussion.

This process included multiple follow up workshops and meetings where the diagrams were refined and system dynamics and relationships were identified, discussed, and refined. There were 2 or 3 follow up half-day workshops to refine each system map, depending on need. System dynamics and relationships were identified, discussed, debated and defined. Notes were taken throughout workshops but due to the confidential nature of discussions and workshop format, digital recording and verbatim transcription was not possible.

### Phase 2—analysis of system maps to identify common themes and functions

The workshops from phase 1 elicited a series of complex system maps looking at peer-led programs and leadership with communities of gay and other men who have sex with men, people living with HIV, people who inject drugs, and sex workers. These included programs engaging at different levels of the HIV and hepatitis C responses in Australia—from frontline peer service delivery, to peer health promotion, and peer leadership. The final versions of the maps and their full descriptions are available as Supplementary Materials, and further updates on the project can be found online at www.w3project.org.au.

As our goal was to develop a framework for monitoring and evaluation that could be applied to peer led programs at different levels and contexts, we needed to analyse and generalize across these system maps. Within the tradition of realist evaluation and synthesis, Pawson and Tilley (47) recommend using abstraction to facilitate the accumulation of knowledge and generalization of findings across cases. We achieved this by asking a theoretical question from ecological resilience theory (51). Envisioning the program as an organism and its community and policy contexts as its surrounding ecology, we asked what functions it would need to be equipped with in order to learn about and adapt to changes in its environment. We analyzed the maps for functions that occurred across all the system maps and incorporated the relationships and flows within the system maps.

We identified four key functions which a peer based program must be able to fulfill in order to be effective and sustainable as it mediates between continually changing community and policy environments.

### Phase 3—review and refinements by organizations locally and validated nationally

**Figure 1:**
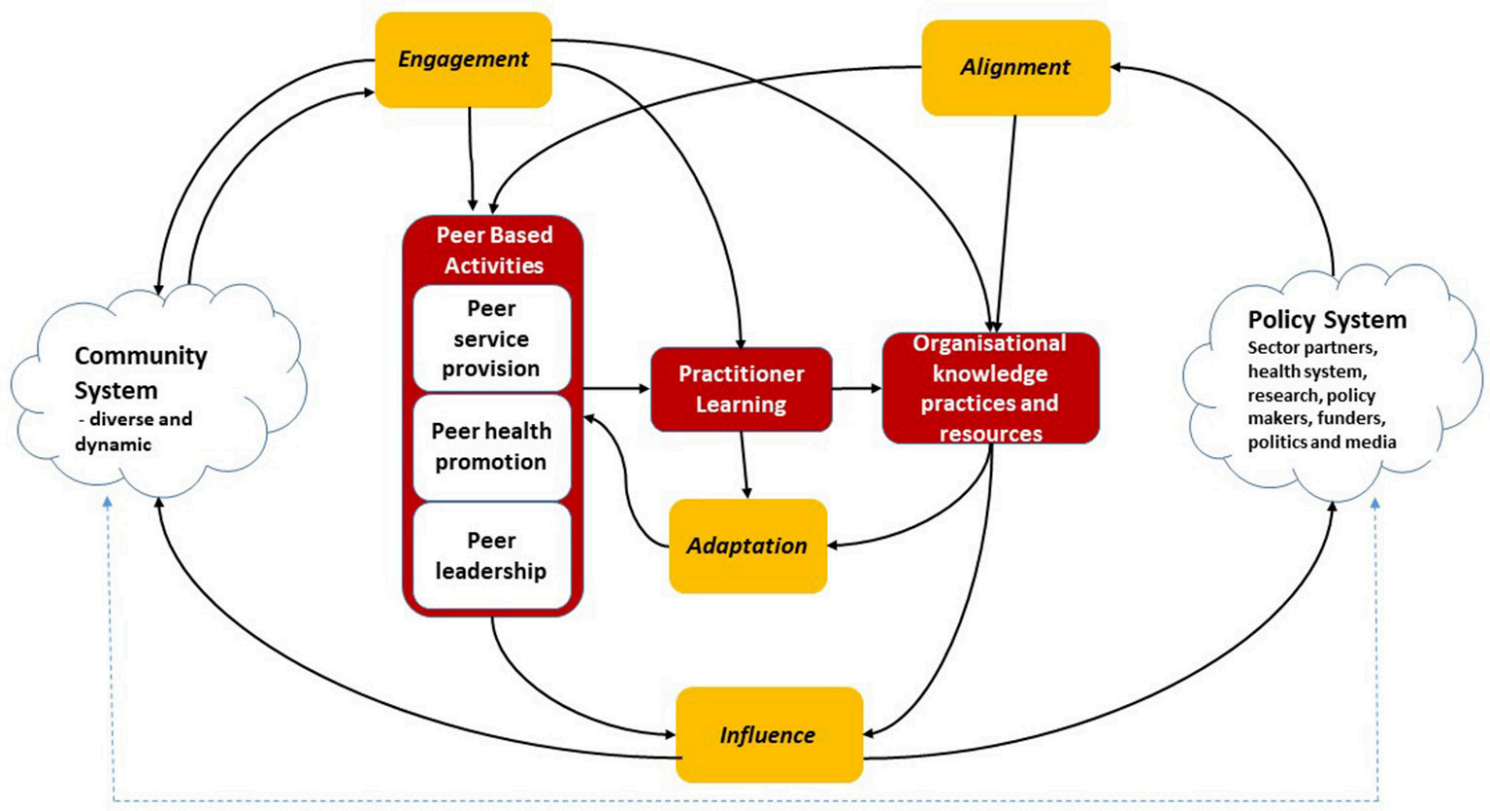
W3 Framework Image. Reprinted from Brown and Reeders (45) under a CC BY license, with permission from (Australian Research Centre in Sex Health and Society La Trobe University), original copyright (2016).

The draft framework was presented, discussed and refined with the partner organizations and then further validated through workshops with non-partner organizations and stakeholders at five national HIV sector meetings and conferences in Australia in 2015–2016. Figure 1 shows the final W3 framework, illustrating the relationships between the four functions.

### Phase 4—developed and tested quality and impact indicators

Using the W3 Framework we then worked with peer organizations to develop tailored indicators for the role, quality and influence of, among other things, peer leadership activities in their system.

More detail about the methods can be found on our website (www.w3project.org.au).

## Results

### Peer leadership in the W3 framework

We found that the peer-led programs were operating within and between two interrelated and constantly changing systems–the community system and the policy (or sector) system. We found there were four functions that were required for peer-led programs to be effective and sustainable in such a constantly changing environment.

- Engagement: How the program maintains up to date mental models of the diversity and dynamism of needs, experiences and identities in its target communities.
- Alignment: How the program picks up signals about what's happening in its policy/sector environment and uses them to better understand how it works.
- Adaptation: How the program changes its approach based on mental models that are refined according to new insights from engagement and alignment.
- Influence: How the program uses existing social and political processes to influence and achieve improved outcomes in both the community and the policy/sector (45).

These are illustrated in Figure 1 and its elements described in Table 2

**Table 2:** Elements and functions of the W3 framework.

**Element**	**Definition**
Community system	The social networks and cultures the program engages with, and the processes of interaction and change that are taking place within them.
Policy system	The policy system includes funders, policy-makers, media, health services, research, and other organizations in the sector.
Peer based activities	Different kinds of peer based approaches that depend on peer skill – the ability to combine personal experience and real-time collective understanding to work effectively within a diverse community
Practitioner learning	Peer workers pick up insights from clients and contacts, and develop, test and refine mental models of their environment.
Organizational knowledge practices	Program management encourages the discussion and capture of insights from practitioner learning as an asset for the organization and for sharing with stakeholders in the policy system.
Arrows	Flows of knowledge or causal influence that constitute the program as a system.
**System level functions**	**Definition**
Engagement	How the program maintains up to date mental models of the diversity and dynamism of needs, experiences and identities in its target communities.
Alignment	How the program picks up signals about what's happening in its policy system and uses them to reorient service or advocacy priorities.
Adaptation	How the program changes its approach based on mental models that are refined according to new insights from engagement and alignment.
Influence	Community How the program uses the community's existing ways of doing things to promote new ways of doing things. Policy How the program achieves or mobilizes influence on processes and outcomes within its policy environment.	

While there are findings and implications from the W3 Project that are specific to different types of peer-led activities (e.g., peer service delivery, peer health promotion, and peer leadership) and to different key populations (e.g., gay men, people who use drugs, sex workers, and people living with HIV), these differences will be explored in detail in forthcoming papers. This paper will focus on broad lessons and implications for peer leadership programs engaging with diverse communities and policy settings.

#### Engagement and peer leadership

Engagement refers to the unique relationship a peer-led program and leadership has within its community, such as the ownership a community feels toward the program, the drawing of staff and volunteers from the community who continue to be part of that community, and the organizations participation as a part of the community. Our findings suggest a broader focus on engagement as a quality of all the combined activities of an organization or program, rather than a specific type of activity (such as targeted consultation). The “community system” is constantly changing, and even though staff and peer advocates may be drawn from it, no one person ever has oversight of all the ways in which change is occurring. Thus, experienced peer workers who participated in our project emphasized knowing the limits of their knowledge

The W3 framework looks for signs of genuine, high quality and sustained engagement that has a purpose: eliciting accurate and real-time community insights to constantly test and update shared mental models about the community in its diversity and dynamism. This includes insights about the role and influence of the peer-led programs within their community.

A key finding from the W3 project is that knowledge generated through high quality engagement can flow, through effective practices of peer leadership, to other policy actors and stakeholders in the prevention sector. This knowledge may identify emerging trends and issues well in advance of formal surveillance and social/behavioral research studies, and this can be crucial in fast-changing contexts. For example, responding quickly to address barriers to safe injecting posed by changing policing practices in street drug markets, or the way PrEP may be increasing or decreasing stigma as the community's engagement with PrEP evolves, or the ways sex workers adapt to changes in policing policies or laws to try and maintain their safety. These real-time insights, and the capacity to identify emerging trends and issues within a community, are critical to peer leadership being relevant and persuasive in the community and policy systems.

#### Alignment and peer leadership

Alignment is similar to engagement, but focuses on picking up signals about changes within the broader policy context of a peer-based program (including policy makers, health services, politics and the media, epidemiology and social research). The broader policy system often presents a complex mix of enablers and barriers that impact on the capacity of a peer-led program to participate and maintain influence within communities. The signals picked up through the alignment function provide guidance to peer leaders and peer-based programs on what adaptations and advocacy may be required. Tackling stigma is an example. While a community driven response to HIV or HCV stigma is essential, it can be impeded by a lack of policy and sector response to the structural factors that can sustain stigma (12). Through alignment, peer leadership may identify the disjunction between stated priorities and what actually gets funded (or supported).

For peer leadership activities to be effective, there needs to be commitment from the broader sector to a community based response to HIV and HCV and the value of considered input from peer leadership. While this commitment can be strengthened by peer led programs demonstrating the outcomes of their peer leadership activities (see Influence below), the commitment it is under constant challenge by rationalization of health funding, policy and legislative frameworks, and the political and social climate. It is not enough to state a commitment to a community based response in a policy document—the response as a whole, including policy-makers, healthcare providers, and researchers, need to adopt practices that support and value the input and contribution that peer leadership can make. The capacity of the peer programs and their organizations to fulfill their peer leadership role was found to be not only an issue of the quality of their engagement with their community, but also alignment from the policy sector to actively value and support the peer organizations to build and sustain this peer leadership capacity. The W3 framework look for signs that peer-based programs are able to identify and anticipate policy, sectoral and health system changes as well as new developments in social, epidemiological, biomedical and implementation research, and then feed them into the next function, Learning and Adaptation. The framework looks for evidence the peer program is able identify the implications, assess the impact of their own advocacy efforts and decide if a different approach is needed. The framework also looks for evidence of the policy and sector system valuing and supporting the peer organizations to develop and maintain an effective peer leadership role within the system. The function of alignment is the key mechanism through which overall synergies among the different components of the prevention system can be achieved.

#### Learning and adaptation and peer leadership

Learning and adaptation includes all the activities through which insights acquired through Engagement and Alignment are shared, discussed, and turned into changes in policy, procedures and work practices within the organization or shareable knowledge outside the organization. Peer practitioners (staff or volunteers) are in a unique position to notice cues and patterns in their community and so constantly update their understanding of how their own personal experience relates to a broader collective understanding of their community. We found that peer leadership activities drew on what we called “peer skill”–the ability to combine their personal experience with a broader collective understanding of their community and use this to work effectively within a diverse community. Systems approaches remind us that not only are things constantly changing—but changes are often emergent and not always predictable. Therefore, peer programs need to draw what they can from engagement and alignment, but then “test the waters” as they adapt with their communities. This includes adaptation within and across the range of peer activities. However, the knowledge from all these experiences of adaptation and learning need to be shared and discussed within the program, and not remain embedded within the experience of individual peer workers. For example—much can be learnt about the evolving nature of stigma in a community through developing and conducting a community led anti-stigma campaign—but only if on-the-ground knowledge is valued and shared. For real-time insights to be effective, the organization within which the peer leadership is based also needs to learn and support continuous and rapid adaptation. This includes ensuring peer leadership activities and advocacy priorities reflect the diversity within their communities, and balances the simultaneous roles of leading and being led by their communities. Therefore, the W3 framework looks for signs of programs learning from insights and adapting with (and sometimes pre-empting) their communities and policy systems.

#### Influence and peer leadership

Within the W3 framework, influence refers to how effectively the program is able to:

- use the community's existing ways of doing things to promote new or adapted ways of doing things; and
- achieve or mobilize influence within its policy and sector environment to enhance processes and outcomes.

The quality of this function is essential to peer leadership. To be influential, peer undertaking peer leadership activities draw on their peer skill and legitimacy as well as a collective and up-to-date perspective of the diverse and changing needs and priorities of their communities. This knowledge must then be “packaged” in a way that is timely and useful for the policy system as well as their communities. In doing this, peer leaders are often required to navigate sometimes contradictory demands and forms of accountability in both the community and policy systems.

Influence within community systems: Peer leadership involves working within, rather than intervening upon, community. In addition to its cultural relevance, it has the credibility of being accountable to community members and groups. The role of peer leadership includes navigating and participating in community debate with its tensions and challenges. Effective peer leadership brings to the strategy an authenticity and credibility based within a long term relationship within the target communities, yet its influence on the community is also drawn in part from the visibility of its advocacy within the policy system and the broader mainstream society. The W3 Framework looks for signs that community members see the peer led program as culturally credible and authentic and expects the peers taking on leadership activities to draw on insights based in part on their own lives. The evolving ways that the community engage with the peer program, and become more empowered, can be an indicator of the authenticity, relevance and impact of past influence.

Influence within policy systems: This refers to the relevance and impact of the contribution made by peer leadership activities within the broader “policy system” that surrounds the public health response to HIV and hepatitis C, including policy-makers, health services, politics and the media, epidemiology and social research. Other organizations in the policy and sector system, as they also adapt to changing environments, can be enablers or barriers to peer leadership achieving its full potential. The way stigma toward gay men who use PrEP or people who use drugs is challenged or tolerated by other services can result in synergies or cross referrals being leveraged or ignored. In some cases, insights from peer led programs may be the sector's only source of real-time knowledge about emerging issues or unintended consequences in rapidly changing environments. A key element of structural stigma is reflected in whether these inputs are valued or discounted. At the same time, however, these insights need to be drawn together in a way that is useful for the sector, and this depends on the quality of peer-led programs' function of alignment. For example, real time insights about how networks of gay men are adapting and responding to the emerging use of PrEP are potentially relevant for policy-makers and health services, as well as to inform plans for in-depth social research. W3 framework looks for signs that peer leadership activities and peer leaders are able to: draw on high-quality engagement to identify key insights about emerging issues; package these in a way that demonstrates their understanding of the pressures faced by other actors in the policy system; and develop a reputation of credibility over time so that policy-makers and sector partners come to trust and act confidently on their input.

### Relationships between functions and other system elements

The most important part of the framework, and what differentiates it from past work in monitoring peer-led programs and leadership, is the focus on understanding how the functions and other system elements interact with each other. The arrows in the W3 Framework (Figure 1) show the flows of knowledge and influence that enable the program to be effective and sustainable in its environment.

The “biomedical revolution” in prevention has triggered rapid social and cultural changes in communities affected by HIV and HCV, despite differences in their access to the medications involved (7–9). This provides an illustration for the W3 framework. Delays in the Australian policy system response to PrEP led to the emergence of informal, activist-led initiatives using social media to facilitate the private importation of generic medication for use as PrEP (12). Peer-led community organizations in Australia have engaged supportively with these initiatives, drawing on their experience and expertise at community forums and in policy advocacy for public funding of PrEP (52). The W3 framework can be used to demonstrate how feedback loops emerge in practice. The quality of peer-based programs' engagement (with community access initiatives) flowed through to increased quality of influence on the policy system, which enhanced their credibility and influence within the community of PrEP users, resulting in increased willingness of this community to engage with the peer-led programs in future.

The flows between the peer program and its policy environment occurs simultaneously. The more peer leaders are able to demonstrate informed and in-depth understanding of the changing needs and practices in their community (engagement), and effective use of peer skill in interpreting research and the experiences of other stakeholders within the policy system (alignment), the more confidence the sector may have in the accuracy and usefulness of their advice (influence). However, if the policy system does not value the role and contribution of peer leadership, regardless of its quality, then this would limit the ability of peer-led programs to demonstrate their effectiveness to the community, and over time, result in a spiral of reduced community willingness to engage and, accordingly, reduced insights into the changing needs and experiences of the community, both for the program and the sector as a whole.

Understanding the complexity and interrelatedness of these dynamics as part of the program theory for peer-led programs provides an opportunity to rethink quality and impact indicators for peer-led programs and leadership.

### Developing practical indicators

An emphasis of the W3 Project's work with our partner organizations was to turn this systems understanding of peer-led programs and leadership into something practical to better monitor and demonstrate the role of peer-led programs. We used the functions in the W3 Framework as headings to develop tailored indicators for each programs quality and impact. We found that it was possible to identify variables at many different points in a causal loop or dynamic in the system, and these variables were prime candidates for the development of quality and impact indicators. We could then identify which of these indicators were most important for a peer-led organization to monitor within its resources and practice.

For example, a program that is influential and valued in its community should be hearing and seeing through its engagement how the community is adapting or responding to the changing environment. A program that is not privy to such changes may be an indicator that it is not reaching or influential within those parts of the community. A program that is not adapting may be an indicator of a program with low community engagement, or a whose policy environment is not allowing it to adapt in tandem with its community, rather than a program maintaining fidelity to an intervention protocol. Therefore, process data on changes in the way the community is engaging with the peer program may provide insight into the quality and impact of their previous community influence, as it is part of the same system dynamic.

Table 3 provides examples of quality indicators for peer leadership drawn from the broader work with the peer-led organizations. The indicators were developed in consultation with partner organizations and serve as examples of check list items to ensure the preconditions for peer leadership and influence in the community and the policy system are being met. The intention is the quality indicators in Table 3 would be tailored to the specifics of a peer program or peer organization, and so be more specific and measurable. The W3 project is now undertaking this process by using the W3 framework to trial tailored indicators and tools for use within specific peer-led organizations. This includes indicators and tools for use in peer-outreach supervision meetings, program planning and review sessions, collation of process and impact evaluation data, and participation in peer-leadership advocacy coalitions and collectives. The focus is on using current process, quality and impact data in a more effective and meaningful way. For further details see www.w3project.org.au.

**Table 3:** Selection of peer leadership quality and influence indicators.

**W3 framework function**	**Example indicators that this function is operating effectively**
Engagement	Program is hearing new things reflective of a community changing and evolving The peer program updates a mental map of the networks and cultures within the community and works to extend its reach within them Community members recognize the program as a participant within its networks and cultures and feel a sense of ownership around its work Peer leaders use personal experience as well as cultural knowledge to communicate and work effectively with community Increased willingness of community to engage in sector consultation and leadership opportunities The peer program identifies emerging practices and unintended consequences of changes in policy or services Peer leadership activities collect and share stories of success to sustain the broader momentum.
Alignment	Peer leaders actively seek out and use knowledge from partners and stakeholders with different perspectives on emerging issues within the sector Other sector stakeholders adapt their approach to support the effectiveness of the peer program. Policy system demonstrates it values and supports the peer leadership role of the peer program. Peer leaders communicate with sector partners to improve each other's understanding of responses to emerging issues. Peer leaders are made aware of changes to policy or services to assess their implications for the community
Adaptation	The peer program integrates peer insights with knowledge acquired from research, and signals from the policy system to support peer leadership advocacy The peer leaders are able to apply a peer lens to update their mental maps of the community and policy systems and pre-empt the implications of changes in the system Organization supports continual learning within the peer program and facilitate the capture and packaging of knowledge from peer insights as an organizational and strategic asset The program supports members to acquire skills in leadership and policy participation Insights from on the ground peer programs update and strengthen the peer leaders understanding of the diverse experiences and adaptations in the community
Influence	Policy The broader sector and policy system values the peer approach and has trust in the insights it generates Readiness and responsiveness of peer leadership activities to opportunities for policy participation Policy, services and funding environment support (or do not impede) innovative and culturally relevant peer led approaches
	Community Community looks toward peer led programs to provide insights into changing meanings of safe sexual and injecting behavior based in the reality of their shared lives Confident peer leaders are visible in the communities Expanding community influence is reflected in new and diverse networks in the community engaging in peer leadership opportunities

## Discussion

Drawing on a systems thinking approach, the W3 framework provides a new way of articulating the role of peer-led programs and peer leadership activities in a changing HIV prevention landscape, and identifying more useful indicators for the monitoring of quality and impact. Feedback from our collaborating partners highlight the “sense making” role of systems thinking approach. The W3 Framework assisted peer leaders to highlight the relationship between the elements of their system. This included relationship between on the ground peer outreach activities and the role of peer leadership activities in policy reforms. The framework (Figure 1) and indicators (Table 2) assist in identifying the most important relationships within that complexity, and then draw together the formal and informal evaluation data, programmatic insights, and community anecdotes in a way that can be more meaningful and useful. The W3 Framework also highlights the role that the rest of the HIV response needs to play in order to gain the most benefit from investments in peer-led programs. Peer led programs, as part of that investment, need the ability to maintain strong connections with all members of the policy system, and possess the necessary resources to maintain alignment of purpose. Peer led programs need to not only demonstrate authenticity to advocate on behalf of their community—but this authenticity needs to be recognized and supported by the sector and timed to when the opportunity emerges or is created (53, 54). When assessing the value of the investment in peer-led programs, the W3 framework provides a mechanism to consider in the analysis of costs and benefits, the program's broader community and system role.

There are limitations in the broad applicability of our work to date. The framework has been based on the expertise and experience of 90 peer practitioners and leaders from 10 peer led organizations in Australia and may not be generalisable to community and policy systems in other countries. To date the W3 framework has been trialed for feasibility at a program level, but not at a sustained organizational or sector level, or comparing the peer leadership capacity, roles and approaches across organizations. A multi-year trial of the use of the framework has recently commenced.

## Conclusion

Like many areas of social and health policy, such rapid changes being experienced in HIV and HCV will present both challenges and opportunities that will test adaptability and effectiveness of peer led programs and leadership. The lack of capacity to demonstrate their broader role in a public health response, peer-led programs continue to be undervalued. There has been little guidance on how to value investment in, or engagement with, peer leadership activities, or what role the rest of the HIV and HCV response have in ensuring full value from these investments.

The What Works and Why (W3) Project undertook a new approach to develop a practice based theory and framework for peer-led organizations and leadership. We found there were four key functions (Engagement, Alignment, Adaptation, and Influence) which a peer led program needs to demonstrate as it navigates and interacts with complex community and policy systems.

If health policy is committed to strengthening the community systems (42) through which we conduct community HIV and HCV programs, then we need to understand, monitor and enhance the role of peer-led programs and leadership as a key and influential partner. As has been argued previously by Collins et al. “It is time for a paradigm shift in how we think about, plan and finance community-based responses” (20). We believe this argument is relevant beyond HIV and HCV and that a systems thinking approach, such as that which led to the W3 Framework, may support us achieving this.

## Author contributions

GB and DR conceptualized and led the study and facilitated the workshops, formal analysis, framework reviews and refinements. AC and JK participated in the workshops, framework reviews and refinements. AM and DO participated in framework reviews and refinements. All authors contributed to the conception and drafting of the work for important intellectual content. All authors have read and approved the final manuscript.

### Conflict of interest statement

GB has received research funding from Australian Government Department of Health for this research. GB has also received funding from Australian Federation of AIDS Organisations, the peak organization for the community response to HIV in Australia. This funding was unrelated to this study; AC is the Director of the National Association of People Living with HIV Australia; AM was formally the Executive Officer of Australian Injecting and Illicit Drug Users League; JK is the Executive Officer of Scarlet Alliance, Australian Sex Worker Association; DO is the Executive Director of the Australian Federation of AIDS Organizations. The remaining authors declare that the research was conducted in the absence of any commercial or financial relationships that could be construed as a potential conflict of interest.
